# Diabetic Ketoacidosis and COVID-19: Patient profiles from a regional emergency department in Johannesburg, South Africa

**DOI:** 10.4102/jcmsa.v3i1.142

**Published:** 2025-07-11

**Authors:** Hanlie Dreyer, Pravani Moodley, Robert Kieser

**Affiliations:** 1 Wits Division of Emergency Medicine, Faculty of Health Sciences, University of the Witwatersrand, Johannesburg, South Africa; 2 Department of Quality Assurance, BioInformatiCo, Johannesburg, South Africa

**Keywords:** diabetic ketoacidosis, COVID-19, emergency department, mortality, South Africa

## Abstract

**Background:**

This study aimed to describe the clinical features and severity of illness in patients diagnosed with both coronavirus disease 2019 (COVID-19) and diabetic ketoacidosis (DKA) at a South African regional hospital.

**Methods:**

This was a single centre, retrospective, observational study conducted at a regional hospital in Johannesburg, South Africa. Records of patients who presented to the emergency department (ED) between 01 April 2020 to 31 August 2021 and were diagnosed with DKA were reviewed. Both COVID-19 positive and negative patients were included.

**Results:**

Of the 104 patients with DKA, 35 (33.7%) tested positive for COVID-19. Among the COVID-19 positive group, two required ICU admission and two were admitted to the High Care Unit (HCU). Interventions included high-flow nasal cannula (1 patient), intubation (2 patients), and inotropic support (1 patient). Outcomes included one death (2.9%), one patient declining treatment, and one transfer. A statistically significant association was found between the COVID-19 positive group and increased age (*p* = 0.02), as well as a positive association with Type 2 Diabetes Mellitus (DMT2) (*p* = 0.03).

**Conclusion:**

This retrospective study demonstrated that patients with COVID-19 and DKA had a lower mortality rate than previously described. In patients with both COVID-19 and DKA, a significant association with advanced age and DMT2 was noted.

**Contribution:**

Data on the burden of co-existing DKA and COVID-19 in South Africa remain limited. This study contributes by highlighting a lower observed mortality rate and underscores the need for further local research on this patient population.

## Introduction

Coronavirus disease 2019 (COVID-19) was first reported in Wuhan, China in December 2019 and has since been declared a global pandemic. South Africa declared a state of disaster in March 2020. Globally, more than 770 million people have been infected with SARS-COV-2 and more than 7 million COVID-19-related deaths have been reported.^1,2^ Patients with comorbidities such as hypertension, diabetes and cardiovascular disease are predisposed to more severe forms of COVID-19 as well as a higher mortality rate.^3,4^

### COVID-19 and diabetes

Multiple case studies and expert recommendations have been published exploring the bidirectional relationship between COVID-19, hyperglycaemia and diabetes mellitus.^3,5,6,7^ Angiotensin converting enzyme 2 acts as an entry point for SARS-CoV-2 and certain diabetogenic effects of COVID-19 have been suggested, such as beta cell function aggravation, stress hyperglycaemia and abnormal glucose metabolism. SARS-CoV-2 infection may precipitate diabetic ketoacidosis (DKA), a severe complication of diabetes.^8^

### COVID-19 and DKA in South Africa

A study from England highlighted a 6% rise in DKA admissions during the pandemic compared to the period before it, but only 12% of those admitted had a confirmed COVID-19 diagnosis. Furthermore, the study found that 41% of those with pre-existing Type 2 Diabetes Mellitus (DMT2) experienced DKA, while 57% of the patients presenting with DKA were diagnosed with new-onset diabetes.^9^ South African emergency departments have seen many patients presenting with COVID-19 and related complications.^2^ In a case series from Durban, South Africa, there were an increased number of patients presenting with DKA than pre-pandemic. There was also an increased mortality rate in comorbid DKA and COVID-19.^10^ In developed countries, the pre-pandemic mortality rate of DKA was under 1%, whereas in South Africa, it ranged from 6.8% to 9%.^11^

Looking at clinical characteristics, a systematic review published in 2020 noted that the majority of patients with both DKA and COVID-19 had a prior diagnosis of DMT2.^12^ A study conducted in New York City investigated the clinical characteristics of individuals with DKA, making comparisons between those who were positive and negative for COVID-19. The results indicated a correlation between older age and the presence of COVID-19 in DKA patients, with older patients having a higher mortality rate of 57% compared to those without the virus.^8^ In another single-centre study from the USA, 43 patients diagnosed with both COVID-19 and DKA were evaluated, revealing a median age of 52 years and glycated haemoglobin levels of 8% or higher, which corresponded to a mortality rate of 58%.^13^ Additionally, a case series from South Africa looked at 10 patients with DKA and COVID-19, most of whom were younger than 45 years, had an average hospital length of stay of 3.3 days, and a mortality rate of 30%.^10^

### Rationale and objective

To date, there are few studies with small sample sizes describing the relationship between COVID-19 and DKA, therefore there is little consensus on the clinical effect that COVID-19 has on DKA. The objective of this research was to characterise the clinical features and burden of disease in patients diagnosed with SARS-CoV-2 infection and DKA in a regional hospital located in Johannesburg, South Africa.

## Research methods and design

### Study design

This was a single-centre, retrospective observational study. All patients aged 12 years and older (institution-based cut-off for paediatrics), who presented to Tambo Memorial Hospital (TMH) Emergency Department between the period of 01 April 2020 and 31 August 2021, were included. Patients diagnosed with DKA were identified from the emergency department (ED) registers. At the time of presentation, patients were tested for COVID-19 by nasopharyngeal reverse transcriptase polymerase chain reaction assay (RT-PCR).

### Setting

Tambo Memorial Hospital is in the Ekurhuleni region of Johannesburg and manages approximately 200 patients annually in DKA or Hyperosmolar Hyperglycaemic State (HHS). During the COVID-19 pandemic, the hospital converted sections into an Intensive Care Unit (ICU) and High Care Unit (HCU) to accommodate the increased burden of disease. The study period of 1 year and 4 months spans the three periods of peak COVID-19 infection (or three waves) in South Africa. Diagnosis of DKA was made according to the Society for Endocrinology, Metabolism and Diabetes of South Africa (SEMDSA) criteria: glucose > 13.9 mmol/L; acidosis pH < 7.3 or HCO_3_ < 18 mmol/L and ketonuria).^14^ All files that had sufficient data to meet the primary objectives were included in the study. Differentiation between Type 1 Diabetes Mellitus (DMT1) and Type 2 Diabetes Mellitus (DMT2) was based on the clinical records, as the patients were already known to the hospital’s medical outpatient department.

### Data collection

Emergency department registers from 01 April 2020 to 31 August 2021 were reviewed by the primary researcher for patients with DKA. During the study period, 236 patients were treated for DKA as per hospital registers. A total of 105 files were retrieved by hospital clerks using a convenience sampling method over a 5-month period. One file was excluded because ED notes were missing. Patient clinical records were analysed and data captured on an Excel spreadsheet, which was designed with all required parameters. Patients identified were allocated a participant identification number, and no identifying characteristics were saved on the data capture form. Only the primary researcher had access to the password-protected participant linkage form with the corresponding hospital and study number. Only files were included that had information regarding the main objectives: demographics of patients, the burden of disease and factors affecting severity, such as blood gas values and ketones. Files without the required information were not considered for the research.

### Data analysis

The data were analysed using IBM SPSS version 28. Descriptive statistics were calculated for all variables with mean and standard deviation. Data were considered statistically significant if *p* < 0.05. The association between SARS-CoV-2 and DKA, patient demographics, comorbidities and other clinical factors were assessed using the Mann Whitney U and the Kruskal-Wallis tests. Multivariate logistic regression analysis was used to predict the relationships between variables.

### Ethical considerations

Ethics approval to conduct this study was obtained from the University of the Witwatersrand Human Research Ethics Committee of the Faculty of Health Sciences (Clearance certificate: MP 220477). Permission to conduct research was also obtained from the Head of the Emergency Unit and CEO of TMH.

## Results

The files of 104 patients were identified and analysed. Of these, 35 patients were noted to be COVID-19 positive (33.65%). The median age of the patients was 37 years. The majority of the patients were of African descent: 31 out of 35 who tested positive for COVID-19 and 54 out of 69 who tested COVID-19 negative. Irrespective of COVID-19 status, there was a male predominance of 58.65%. Of note, 64 patients (61.53%) had pre-existing DM and 40 patients (38.46%) were diagnosed with Diabetes Mellitus (DM) on admission, irrespective of COVID-19 diagnosis (Table 1). Other notable comorbidities, regardless of COVID-19 diagnosis, included hypertension in 37 patients (36.58%), human immunodeficiency virus (HIV) in 12 patients (11.54%) and dyslipidaemia in five patients (4.81%). The study identified patients with DKA during different infection waves as follows: in the first wave, 16 out of 45 were COVID-19 positive; in the second wave, 4 out of 23 were COVID-19 positive; and in the third wave, 15 out of 36 were COVID-19 positive.

**TABLE 1:** Descriptive characteristics and clinical profile of patients in diabetic ketoacidosis.

Variable	COVID-19 positive (*N* = 35)		COVID-19 negative (*N* = 69)		*p*-value
	*n*	%	*n*	%	
**Age (years)**	-	-	-	-	0.28
< 25	3	8.57	13	18.84	-
25–34	6	17.14	20	28.99	-
35–44	8	22.86	14	20.29	-
45–54	7	20.00	10	14.49	-
> 55	11	31.43	12	17.39	-
**Gender**	-	-	-	-	0.84
Male	20	57.14	41	59.44	-
Female	15	42.86	28	40.58	-
**Race**	-	-	-	-	0.69
Asian people	0	0.00	2	2.90	-
African people	31	88.57	54	78.26	-
Mixed race people	1	2.86	2	2.90	-
White people	3	8.57	11	15.94	-
**Comorbidities**	-	-	-	-	-
Type 1 DM	5	14.29	24	34.78	0.08
Type 2 DM	15	42.85	20	28.99	0.08
Newly diagnosed DM	15	42.85	25	36.23	0.08
Hypertension	16	45.71	21	30.43	0.14
HIV	6	17.14	6	8.70	0.21
Dyslipidaemia	1	2.86	4	5.80	0.66
**Treatment history**	-	-	-	-	0.11
Insulin	5	14.29	25	36.23	-
Metformin	6	17.14	4	5.80	-
Defaulted insulin or oral medication	2	5.71	5	7.25	-
Antiretrovirals	4	11.43	2	2.90	-
Antihypertensives	2	5.71	2	2.90	-
Unknown treatment	4	11.43	4	5.80	-
Multiple agents	4	11.43	11	15.94	-
Not on medication	8	22.86	16	23.19	-

DM, diabetes mellitus; COVID-19, coronavirus disease 2019; HIV, human immunodeficiency virus.

### Associations between COVID-19, age and comorbidities

Multivariable logistic regression analysis showed a significant difference in age between DKA patients who tested positive for COVID-19 and those who tested negative, with COVID-19-positive patients being older on average (95% confidence interval [CI], 44.66 ± 15.18 versus 37.36 ± 14.89, *p* = 0.02). Further analysis of diabetic patients who tested positive for COVID-19 revealed that 5 out of 29 patients had DMT1, while 15 out of 35 had DMT2. This indicated a significantly higher prevalence of DMT2 compared to DMT1 among the patients (17.2% versus 42.9%, *p* = 0.03). No other significant associations were found in this cohort (Table 2).

**TABLE 2:** Multivariate analysis of COVID-19 positive and variables.

Outcome variable	Coefficient *B*	*p*-value	Odds ratio	95% confidence interval
Age	0.03	0.024	1.03	1.00–1.06
Na	0.06	0.045	1.06	1.00–1.12
Type 2 DM	1.28	0.032	3.60	1.11–11.64

Na, sodium; DM, diabetes mellitus.

The biochemical markers on admission were similar in both groups (COVID-19 positive and negative). The mean haemoglobin A1C (HbA1C) was 13.56% and 13.52%; pH: 6.95 and 6.90; osmolarity: 305 versus 302; and anion gap: 21.72 and 22.11, respectively, for COVID-19 positive and negative patients. There was no statistically significant association observed between these parameters (Table 3).

**TABLE 3:** Biochemical markers at admission.

Variable	COVID-19 positive mean		COVID-19 negative mean		*p*-value
	Mean	s.d.	Mean	s.d.	
CRP	76.51	94.73	61.18	103.98	0.09
HbA1C (%)	13.56	2.87	13.52	2.99	0.95
WCC	12.12	5.57	16.00	10.07	0.08
Urea	10.37	5.72	10.30	8.20	0.38
Creatinine	137.86	64.67	153.43	99.54	0.51
pH	6.95	1.22	6.90	1.21	0.15
Glucose	27.65	9.15	29.76	9.61	0.27
Osmolarity	305.96	27.43	302.67	18.23	0.60
Anion gap	21.72	6.27	22.11	6.77	0.78

COVID-19, coronavirus disease 2019; CRP, C-reactive protein; s.d., standard deviation.

### Burden of disease and mortality

**FIGURE 1:**
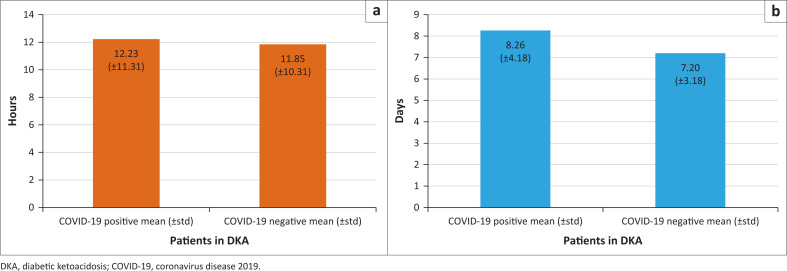
Length of stay: (a) hours spent in emergency department; (b) days spent in hospital.

The mean length of ED stay was 12.23 (±11.31) hours in the COVID-19 positive group and 11.84 (±10.31) hours in the COVID-19 negative group (*p* = 0.22). The mean hospital stay was 8.26 (±4.18) days in the COVID-19 positive group, compared to 7.20 (±3.81) days in the COVID-19 negative group, *p* = 0.22 (Figure 1).

Regarding the level of care, five patients required ICU hospitalisation. Among them, only two tested positive for COVID-19 – a 31-year-old and a 46-year-old – both newly diagnosed with diabetes. The COVID-19-negative ICU patients included two with DMT1 and one who was newly diagnosed. Their ages were 16 years, 27 years and 37 years.

Regarding respiratory symptoms and treatment of the 35 COVID-19-positive patients, only 12 needed respiratory support. The majority experienced mild symptoms, with three requiring nasal cannulas, six requiring face mask oxygen, one requiring high flow nasal cannula and two requiring intubation. Inotropic support was started for one patient. Only one patient from the COVID-19-negative group required intubation for low Glasgow Coma Scale (GCS), which subsequently improved, and the patient was discharged.

Among the COVID-19-positive group, one patient died (2.86%), one patient declined hospital treatment and three patients were transferred to a quaternary facility. No deaths were noted within the COVID-19-negative group.

## Discussion

The exact pathophysiology of the interaction of DKA in COVID-19 is not fully understood, with limited information regarding clinical characteristics and burden of disease.

The bidirectional relationship between hyperglycaemia and COVID-19 has been reported in case studies. This study analysed data from patients presenting in DKA who tested positive and negative for COVID-19.

The objective was to look at clinical characteristics, burden of disease and examine the relationship between COVID-19 and hyperglycaemia, particularly within a South African context.

This study observed a predominance of males (58.65%) and individuals of African ethnicity (81.73%), likely reflecting the characteristics of the underlying population. These results were similar to a small study from Durban, South Africa, with 10 patients which found a male predominance of 60% and African ethnicity of 90%.^10^

A mean age of 44.7 years was noted among COVID-19-positive patients and 37.4 in the COVID-19-negative group. A statistically significant association was found between patients who tested positive for COVID-19 and were older (*p* = 0.02). These findings are similar to the results of a New York study by Patel et al. (mean age 58.6, and COVID-19 patients were older *p* = 0.01).^8^

Our study noted that diabetic patients who tested positive for COVID-19 were significantly more likely to have DMT2 compared to DMT1 (*p* = 0.03), which is consistent with findings from a case series from Durban and the New York City study.^8,10^ Additionally, our study identified 40 patients who were newly diagnosed with diabetes during their DKA presentation, with 15 of them testing positive for COVID-19, accounting for 38%. This contrasts with the England study that noted 7% of patients had newly diagnosed DM with concurrent COVID-19.^9^

Regarding the disease burden, a 2023 study conducted in the United Arab Emirates analysed 11 COVID-19-positive patients with DKA. The study found that these patients had an average HbA1C of 13.5%, which closely matches the HbA1C of 13.56% in our study. HbA1C is used as a marker of treatment compliance in patients with Diabetes Mellitus. The UAE study reported an average C-reactive protein (CRP) of 50, whereas our study found an average CRP of 76. Additionally, the pH average was 7.2 in their study, compared to 6.9 in ours.^15^ The Durban study reported an average pH of 7.02, CRP of 193, HbA1C of 11.9 and an average hospital stay of 3.3 days, whereas our study found a longer average stay of 7.56 days. These differences may indicate an increased severity among our cohort of patients as compared to the UAE and Durban study^10,15^

The mortality rate among patients with DKA and COVID-19 in our study was notably lower at 2.86%, compared to higher rates reported in case studies and a systematic review by Pal et al.,^12^ where patients with COVID-19 frequently presented with more severe DKA, and mortality rates ranged from 18% to 58%.^9,12,13,15^ The systematic review of patients with DKA and COVID-19 had a mortality rate of 45% and noted that a low pH on presentation had a significant association with in-hospital mortality.^12^ The Durban study noted a 30% mortality among patients with concurrent DKA and COVID-19 diagnosis, but this could be attributed to the small study size.^10^ The New York study assessed the baseline characteristics of 35 patients with DKA and COVID-19 and reported a 57% mortality rate, significantly higher than figures reported in other studies. Additionally, the mean age of patients in the New York study was older compared to our cohort, with an average age of 58.6 years compared to 44.7 years in our study.^8^

The low mortality rate noted in this study could be attributed to the time periods investigated and the sample size. In South Africa, the COVID-19 waves were driven by the spread of a new, more transmissible COVID-19 variant. The first wave spanned June 2020 to August 2020; the second wave from November 2020 to February 2021; and the third wave from May 2021 to August 2021.^16^ Regarding the COVID-19 waves documented, we noted the following: 16 out of 45 patients tested COVID-19 positive during the first wave, 4 out of 23 during the second wave and 15 out of 36 during the third wave. This differs from another retrospective South African study, Bhikoo et al. that noted an increased rate of infection and hospitalisation in the second and third waves.^17^

Early during the pandemic, COVID-19 treatment options were based on case discussions and expert opinion with varying treatment strategies suggested.^18^ It could be hypothesised that the longer time frame of our study, ongoing vaccination efforts that had been implemented, increase in healthcare system capacity and improved COVID-19 treatment protocols, could have contributed to a decrease in the mortality rate.^19^

### Limitations

This study has several limitations that must be considered. The sample size was small, with only 35 COVID-19 positive patients out of 104, which limits statistical power and generalisability to larger populations. Additionally, the use of convenience sampling from a single regional hospital may introduce selection bias, reducing the representativeness of the cohort. The disproportionate number of DMT1 patients compared to DMT2 may also skew the results, as clinical features and outcomes could differ between these groups. Incomplete or unavailable data further introduced potential biases, limiting the depth of the analysis. The selection bias introduced by the convenience sampling and the missing records means that our findings may not be generalisable to all patients with DKA and COVID-19 presenting to TMH. It is possible that patients with mentioned specific characteristics, such as more severe illness, longer hospital stays and incomplete records, were underrepresented in our sample. Finally, challenges in accessing patient records due to the public healthcare setting affected sample completeness and data quality.

## Conclusion

Based on our study, the demographics of patients who tested COVID-19 positive and negative with DKA were similar to other studies. We found a significant correlation between COVID-19-positive patients and those with Diabetes Mellitus Type 2 and older age. Patients with DKA and COVID-19 had a lower mortality rate of 2.86% than other studies of similar size which could be due to vaccination efforts as well as improved COVID-19 treatment protocols.

### Dissemination of results

The study results will be shared to participating institutions and disseminated through various emergency medicine social media platforms.
